# A cost–benefit analysis of hospital-wide medication reviews: a period prevalence study

**DOI:** 10.1007/s11096-021-01323-1

**Published:** 2021-09-08

**Authors:** Sarah Wilkes, Rianne J. Zaal, Alan Abdulla, Nicole G. M. Hunfeld

**Affiliations:** 1 Department of Hospital Pharmacy, Erasmus MC, University Medical Center Rotterdam, Rotterdam, The Netherlands; 2 Department of Intensive Care, Erasmus MC, University Medical Center Rotterdam, Rotterdam, The Netherlands

**Keywords:** Cost–benefit analysis, Drug-related problems, Medication reviews, Patient safety

## Abstract

*Background* For specific medical specialties it has been shown that clinical pharmacists can have a beneficial effect on the reduction of drug-related problems by performing medication reviews. However, little is known on the cost–benefit ratio of hospital-wide implementation of medication reviews. *Aim* To investigate the effect of conducting hospital-wide medication reviews on the detection and resolution of drug-related problems, and to calculate the cost–benefit ratio of the intervention. *Method* In this observational prospective period prevalence study, medication reviews were conducted during five consecutive working days in a Dutch university hospital. Patients admitted for more than 24 h were included. The cost–benefit ratio of conducting the medication reviews was calculated by dividing the total costs by the total savings. *Results* In 622 medication reviews, 709 potential drug-related problems (1.1 per patient) were detected. The most common advice was to stop medication (38.6%). Patients with a potentially drug-related problem were significantly older, had a higher median number of prescriptions, and the median number of days from admission to the time of medication reviews was longer. Conducting medication reviews showed a positive cost–benefit ratio of 9.7. *Conclusions* Hospital-wide medication reviews by clinical pharmacists have a positive cost–benefit ratio and contribute to the detection and the resolution of drug related problems (DRPs), mainly by reducing overtreatment.

**Supplementary Information:**

The online version contains supplementary material available at 10.1007/s11096-021-01323-1.

## Impact on practice

- Conducting medication reviews, on top of clinical decision support systems, leads to the detection and resolution of pharmacological overtreatment and undertreatment
- Hospitalized patients who are older, or who use multiple prescriptions are more at risk for drug-related problems
- Conducting hospital-wide medication reviews shows a positive cost–benefit ratio and should therefore be implemented in every hospital

## Introduction

Most patients admitted to a hospital use more than five different drugs [1]. Besides the beneficial effects of these drugs, medication errors occur frequently. In fact, due to the complexity of the medication process, medication-related errors are one of the most common types of error in hospitals [2, 3]. These medication errors represent a significant patient safety concern and are associated with additional costs [4].

Clinical decision support systems (CDSSs) are traditionally used by hospital pharmacists to detect and prevent drug-related problems (DRPs). Although CDSSs are getting more advanced, they are still not able to detect all DRPs. Several studies show that the involvement of a clinical pharmacist conducting medication reviews, besides using CDSS, has beneficial effects on medication safety [5–12].

A clinical pharmacist, as defined by the American College of Clinical Pharmacy (ACCP), works directly with physicians, other health professionals, and patients to ensure that prescribed medication contributes to the best possible health outcomes [13]. In contrast to CDSS, clinical pharmacists can combine information about prescribing guidelines, medical history and laboratory values with the current diagnoses to optimize pharmacotherapy.

Medication reviews conducted by clinical pharmacists have become an integral part of healthcare in many countries [13]. Although no evidence was found that conducting medication reviews reduces mortality, or hospital readmissions, studies showed a reduction in emergency department contacts [14, 15]. Furthermore, a positive impact of the involvement of a clinical pharmacist on the ward was demonstrated for specific patient groups [6, 9], including patients admitted to intensive care units [5, 16, 17]. Because of the beneficial effect on patient safety, there is a shift from the traditional way of practice in which a clinical pharmacist reacts on the signals generated by the CDSS, to a proactive clinical pharmacist integrated in the medical team on the ward. However, little is known about the impact of a hospital-wide implementation of clinical pharmacists who perform medication reviews to improve patient safety and the cost–benefit analysis of this intervention.

### Aim of the study

The aim of this study is to investigate the effect of conducting hospital-wide medication reviews on the detection and resolution of drug-related problems, and to calculate the cost–benefit ratio of the intervention.

### Ethics approval

The study protocol was reviewed by the Erasmus MC Medical Ethics Committee. This committee provided a waiver for obtaining informed consent (MEC-2019-0687).

## Method

### Design and setting

An observational prospective period prevalence study was performed at the Erasmus Medical Center, a 1246 bed university hospital in the Netherlands. Since May 2018, the clinical pharmacists are integrated in the teams on all clinical wards. For this study, the clinical pharmacists on every ward reviewed the pharmacotherapy of admitted patients for a period of five consecutive working days between August 2019 and June 2020. For every patient the potential DRPs were discussed with the physician and registered in the patient record.

In this hospital medication is prescribed using a computerized physician order entry system combined with a CDSS, based on the Dutch national drug database G-standard (Z-Index, The Hague, The Netherlands). Alerts about overdosing, duplicate therapy, drug-drug interactions, allergy’s, contra-indications and omissions are provided for prescribers, pharmacy technicians and pharmacists. The pharmacy technicians and pharmacists receive additional alerts about performing therapeutic drug monitoring, dosage adjustment needed by reduced renal function and the combination of low molecular weight heparins with vitamin K antagonists. Interventions based on signals generated by the CDSS were excluded as DRP in this study. The clinical pharmacists were trained by a clinical pharmacist (SW) before conducting the medication reviews. All clinical pharmacists were trained as a hospital pharmacist and had at least two years of experience as a clinical pharmacist. The hospital pharmacy residents received at least three years of training to become a hospital pharmacist and were supervised by a clinical pharmacist.

A DRP is defined as an event, or circumstance involving drug therapy that actually, or potentially interferes with desired health outcomes [18]. In the current study, a potential DRP (pDRP) was defined as a DRP that was detected by a pharmacist, but was not yet discussed with the physician. If the physician agreed with the pharmacist that the pDRP was relevant for the patient, the pDRP was changed into a DRP.

### Primary endpoint

To determine the prevalence of pDRPs per patient after a hospital-wide implementation of medication reviews and to calculate the cost–benefit ratio of this intervention.

### Secondary endpoints

To describe the severity of the DRPs, the types of recommendations and their acceptance by the physician.

### Inclusion and exclusion criteria

During the study period of five consecutive working days on a ward, patients of all ages admitted to that specific ward were eligible for inclusion. An overview of the clinical departments that participated, is presented as supplementary information (Table S1). Patients admitted to the hospital with an expected length of stay less than 24 h were excluded.

### Medication review

Clinical pharmacists conducted medication reviews according to the definition of a medication review as stated by the Pharmaceutical Care Network Europe [19]: “Medication review is a structured evaluation of a patient‘s medicines with the aim of optimising medicines use and improving health outcomes. This entails detecting drug-related problems and recommending interventions.”

For every patient the medication was evaluated on the following topics: optimal pharmacotherapy for the diagnosis according to the recent guidelines, laboratory values in combination with drugs (e.g. renal function), drug-drug interactions, dosage, contra-indicated drugs, drug use problems, indication for therapeutic drug monitoring, medication reconciliation, adverse drug reactions, drug allergies, and correct registration of the medication order. The detected pDRPs were discussed with a physician.

### Data collection

The following information was documented in a standardized database: patient characteristics, drug classes according to Anatomical Therapeutic Chemical (ATC) code, the total amount of interventions, type of interventions, type of recommendation, time spent per review, follow up of recommendations by the physician within 24 h and the way of communicating the recommendation.

### Severity of drug-related problems

To classify the DRPs, the National Coordinating Council for Medication Error Reporting and Prevention (NCC MERP) Index was used [20]. This index consists of nine categories (A–I) that are further combined into four categories, based on the severity of the outcome: (1) no error (A), (2) error, no patient harm (B–D), (3) error, patient harm (E–H), and (4) error, death (I), see Table 1. The risk assessment was done by the clinical pharmacist that detected the DRP. Afterwards an independent clinical pharmacist reassessed the score. After double assessment, the two pharmacists discussed any discrepancy in the severity score to reach consensus. In case the two assessors could not reach consensus a third pharmacist was consulted to reach consensus.

**Table 1:** NCC MERP index and Nesbit score for categorizing medication errors [20, 21]

Category NCC MERP		Content	Nesbit probability score	
No error	A	Circumstances that have the capacity to cause error	0 = zero	Information only
Error, no patient harm	B	An error occurred, but the error did not reach the patient		
	C	An error occurred that reached the patient, but did not cause patient harm	0.01 = very low	Problem orders
	D	An error occurred that reached the patient and required monitoring to confirm that it resulted in no harm to patient and/or required intervention to preclude harm	0.1 = low	Some harm is expected, but poorly clinical relevant
Error, patient harm	E	An error occurred that may have contributed to, or resulted in temporary harm to the patient and required intervention	0.4 = medium	Harm is expected, clinically relevant
	F	An error occurred that may have contributed to, or resulted in temporary harm to the patient and required initial, or prolonged hospitalization	0.6 = high	Harm is expected, life threatening
	G	An error occurred that may have contributed to, or resulted in permanent harm		
	H	An error occurred that required intervention necessary to sustain life		
Error, death	I	An error occurred that may have contributed to, or resulted in the patient’s death		

### Cost–benefit analysis

The cost benefit ratio of the intervention was calculated by dividing the total costs by the total savings (cost avoidance summed with cost savings). This cost–benefit ratio was expressed for the intervention period of five consecutive working days.

### Cost avoidance and cost savings

To calculate cost avoidance, only the accepted DRPs were included. To estimate the probability that in the absence of the DRPS an adverse drug event (ADE) would occur, the Nesbit probability score was used [21]. The probability of the occurrence of an ADE in the absence of a DRP was set at a likelihood of an ADE of 0 (zero), 0.01 (very low), 0.1 (low), 0.4 (medium), or 0.6 (high). The NCC MERP categories were matched with the Nesbit probability score (Table 1). As cost price for an ADE we used € 1098.88. This is based on a cost study of ADEs in a German hospital [22] and adjusted to standard inflation to match the costs in 2020 [23].

The Nesbit probability scores were multiplied with the cost of an ADE to measure cost avoidance. To calculate cost savings, the costs of discontinued medication was calculated, using the Dutch medication price list [24]. The daily drug costs were multiplied by the number of days until hospital discharge, with a maximum of five days.

### Costs of intervention

The direct labor time of the pharmacists was multiplied by the costs of a clinical pharmacist per hour. In the Netherlands this is € 82.50 per hour in a university hospital.

### Data analysis

All data analyses were performed using IBM-SPSS (version 25.0, IBM Corp., New York, NY, USA). Categorical variables were expressed as frequencies (percentages), and continuous variables were described as median values with range. To identify the differences between patients with a pDRP and without a pDRP after medication review, chi-square tests were performed for nominal data. Continuous variables were analyzed with a Mann–Whitney U test. A p-value *p *< 0.05 was considered as statistically significant.

## Results

Overall, for 558 patients 622 medication reviews were conducted by 14 hospital pharmacists and 3 hospital pharmacy residents. In total, 20 interventions were excluded from the analysis, due to inconclusive data (16), the DRP was detected by the CDSS instead of the reviews (1), the DRP was detected and communicated before conducting the medication review (1), or the patient discharged within 24 h (2).

### DRPs

A total of 709 pDRPs, 1.1 pDRPs per patient, were detected by the clinical pharmacist in 51% (320) of the medication reviews. 479 (67,6%) recommendations were accepted by the attending physician and given follow up within 24 h. The patient characteristics are summarized in Table 2. Patients with a pDRP were significantly older, had a higher median number of prescriptions, and the median number of days from admission to the time of medication reviews was longer. The average time spent per medication review was 8.9 min.

**Table 2:** Patient characteristics

		Medication review with a pDRP (%)	Medication review without a pDRP (%)	Statistics
Patients		316	306	
Gender	Female	137 (43.4)	147 (48.0)	*p *= 0.241
Age				***p *< 0.05**
	0–12 months	23 (7.3)	47 (15.4)	
	1–12 years	7 (2.2)	16 (5.2)	
	13–18 years	10 (3.2)	13 (4.2)	
	19–40 years	30 (9.5)	80 (26.1)	
	41–60 years	87 (27.5)	68 (22.2)	
	61–80 years	142 (44.9)	74 (24.2)	
	> 80 years	17 (5.4)	8 (2.6)	
Prescriptions, median [range]		13 [1–31]	8 [0–24]	***p *< 0.05**
	None	0 (0)	14 (4.6)	
	1–5	24 (7.6)	92 (30.1)	
	6–10	90 (28.5)	107 (35.0)	
	10–15	95 (30.1)	52 (17.0)	
	> 15	107 (33.9)	41 (13.4)	
Elective admission		127 (40.2)	124 (40.5)	***p*** = **0.933**
Day after admission, median [range]		5 [0–241]	3 [0–155]	*p* = **0.044**

*pDRP* potential drug-related problem. The numbers in bold are statistically significant

**Fig. 1:**
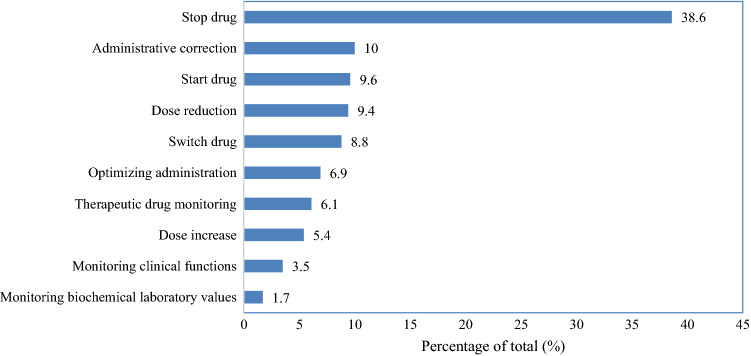
Type of the recommendations given by the clinical pharmacist with follow up within 24 h (n = 479)

**Fig. 2:**
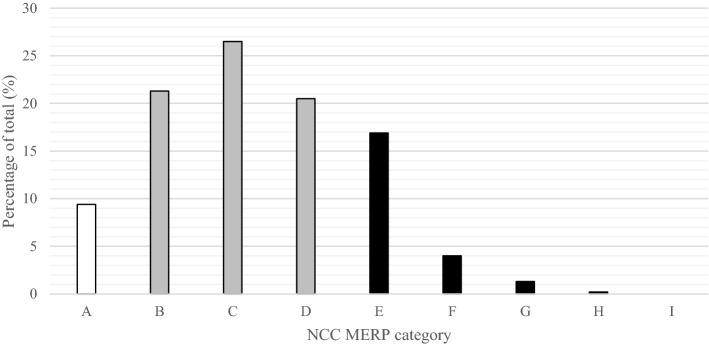
The severity of the drug-related problems [20] that were given follow up within 24 h. White: category no error, grey: category error, no patient harm and black: category error, patient harm

Drug use without indication (26.9%), administrative prescribing errors (19.8%) and drug omission (12.5%) were the most common detected DRPs (Table 3). The most frequently given recommendations were to stop medication (38.6%), to make an administrative correction of the medication order (10%) and to start medication (10%) (Fig. 1). Most DRPs were categorized as relevant problem without patient harm (Table 4 and Fig. 2).

**Table 3:** Detected drug-related problems, with a clinical example, that were given follow up within 24 h

	n	%
Drug use without indication	129	26.9
*e.g. advice to stop metoclopramide since it was no longer indicated*		
Administrative prescribing error	95	19.8
*e.g. the dosage is described as ‘dosage known by the patient’ instead of the actual dosage*		
Drug omission	60	12.5
*e.g. the omission of statin therapy*		
Incorrect dosage	43	9.0
*e.g. dose adjustment of vancomycin due to the start of hemodialysis*		
Drug use problem	38	7.9
*e.g. the patient was unable to swallow the prescribed medication*		
Monitoring of the patient needed	35	7.3
*e.g. advice to measure the renal function during treatment with nonsteroidal anti-inflammatory drugs (NSAIDs)*		
Duplicate therapy	29	6.1
*e.g. simultaneous use of amlodipine and barnidipine*		
Other	25	5.2
*e.g. thyroid therapy was not taken on an empty stomach*		
Allergy, or contra-indication	15	3.1
*e.g. the omission of the registration of the Brugada syndrome as contra-indication*		
Side effect	6	1.3
*e.g. elevated creatinine kinase (CK) levels as side effect of ciprofloxacin*		
Drug-drug interaction	4	0.8
*e.g. the manual check on drug interaction with cannabinoids*		
**Total**	479	100

**Table 4:** The severity of the drug-related problem [20], including a clinical example, that were given follow up within 24 h

		Total			
Category		n	(%)	n	(%)
	**No error**			**45**	**9.4**
A	Circumstances that have the capacity to cause error	45	9.4		
	*e.g. omission of an end date for antibiotic treatment while the end date is currently unknown. The physician did however make a note to register the end date in the future*				
	**Error, no patient harm**			**327**	**68.3**
B	An error occurred, but the error did not reach the patient	102	21.3		
	*e.g. two types of parental nutrition were prescribed, only one was administered to the patient*				
C	An error occurred that reached the patient, but did not cause patient harm	127	26.5		
	*e.g. intravenous administration of a proton pump inhibitor whereas oral administration is possible for the patient*				
D	An error occurred that reached the patient and required monitoring to confirm that it resulted in no harm to patient and/or required intervention to preclude harm	98	20.5		
	*e.g. the need for therapeutic drug monitoring of levetiracetam, indicated because of the impaired renal function*				
	**Error, patient harm**			**107**	**22.3**
E	An error occurred that may have contributed to, or resulted in temporary harm to the patient and required intervention	81	16.9		
	*e.g. continuation of metformin, ACE inhibitor and spironolactone in a patient with acute renal failure*				
F	An error occurred that may have contributed to, or resulted in temporary harm to the patient and required initial, or prolonged hospitalization	19	4		
	*e.g. the overdose of a low molecular weight heparin*				
G	An error occurred that may have contributed to, or resulted in permanent harm	6	1.3		
	*e.g. the omission of anticoagualation therapy in a patient with atrial fibrillation*				
H	An error occurred that required intervention necessary to sustain life	1	0.2		
	*e.g. the omission of antimycotic therapy in an ICCU patient*				
	**Error, death**				
I	An error occurred that may have contributed to, or resulted in the patient’s death	0	0		
	Total			**479**	**100**

The drugs that were most commonly involved with a DRP were antibiotics for systemic use (15.9%), drugs for acid related disorders (7.7%), analgesics (7.7%) and antithrombotic agents (6.7%). 59.9% of the DRPs were related to drug therapy that was started during hospital admission.

The pDRPs were most often discussed face to face with the physician (62.2%). These were also communicated by phone (22.3%), by making a note in the electronic patient record (10.4%), or by email (4.8%).

### Cost–benefit analysis

The cost of service was based on 92.3 direct labor hours, leading to €7611.73 (A) per week. After adjusting for the Nesbit probability score, 59.07 ADEs were prevented, resulting in a cost avoidance of €64,910.84 (B). The total amount of cost savings was €8659.54 (C) per week. This shows a positive cost–benefit ratio of 9.7 (B + C/A).

## Discussion

We show that a hospital-wide implementation of medication reviews by clinical pharmacists results in the detection of 1.1 pDRP per patient. Drug use without indication and the omission of drugs were frequently detected DRPs, resulting in drug initiation and discontinuation as most common recommendation. Besides the beneficial effect on drug safety these interventions showed also a positive cost–benefit ratio of 9.7.

To our knowledge, this is the first study to demonstrate that hospital-wide medication reviews have a beneficial effect on drug safety on patients of all ages in a university hospital. In the Netherlands, the effect of clinical pharmacists conducting medication reviews was previously studied on surgical and neurological wards [9], internal wards [6], and the intensive care unit [5]. In these studies, pDRPs were detected in 23–76% of the medication reviews [5, 6, 9], which is in line with our findings (50%). Furthermore, the recommendation to stop, or start medication was also among the most common recommendations found by Zaal et al.[9] and Bosma et al.[5, 6]. In contrast to our design, these studies focused on specific wards. Our detection rate of pDRPs and the most frequently given recommendations are also in line with previous studies on hospital-wide integration of clinical pharmacists in other hospitals worldwide [11, 12, 25–27].

Signals about dose adjustment and duplicate drug therapy are mostly generated by the CDSS and therefore less frequently reported in this study. The high amount of administrative prescribing errors in our study might indicate that physicians need more training for adequate prescribing skills, or that the prescribing system might not work intuitively to prevent this type of error*.* Poor prescribing practice was also found by Ronan et al.[11], where administrative prescribing errors accounted for 15% of the DRPs.

The drugs that were most commonly related to a DRP were antibiotics for systemic use (15.9%), drugs for acid related disorders (7.7%), analgesics (7.7%), and antithrombotic agents (6.7%). This is in consistence with the findings in the literature [11, 12, 25, 26, 28].

Most of the suggested interventions (67.7%) were given a follow up within 24 h by the attending physician. This is in line with the acceptance rate in previous studies (56–88.5%) [5, 6, 9, 11, 28–30]. However, in the literature the time allowed to accept the intervention varied between 24 and 72 h.

The average time spent per medication review was 8.9 min. Bosma 2008 et al. [6] described an average time spent of 50 min per patient. However, they also included the actual participation in the physicians rounds, while our study did only register the actual time spent on conducting the medication review. However, even if the average time spent on the review is five times longer, the cost–benefit ratio still remains positive.

If time is limited for conducting medication reviews, it is sensible to start with patients at risk for medication errors. Our data shows that pharmacists detect a pDRP more often if the patient was older, had more prescriptions and the review was not conducted directly after admission. While not within the scope of this study, it would be of interest for future studies to a priori identify at-risk patients in need of medication reviews. This will be in favor of the cost–benefit ratio, but more importantly, at-risk patients will receive the necessary attention and the workload for hospital pharmacist will remain limited. To further reduce the workload, the CDSS needs to be optimized by implementing signals for omissions in drug therapy and drug therapy without indication.

One of the strengths of this study is the hospital-wide implementation of the medications reviews, resulting in inclusion of patients of all ages and comorbidities. Another strength is that only the pDRPs based on the medication review were reported, and not the pDRPs detected by the CDSS. In this way the additional value of the clinical pharmacist was studied, since the use of CDSS is common practice. Also, we showed that the hospital-wide implementation of medication reviews has a positive cost–benefit ratio, which is an important element for policymakers in the hospitals. To reduce the costs of overtreatment we advise to focus on high priced drugs, since 10% of the drugs accounted for 75% of the cost savings.

This study has several limitations. First, we collected the data for a the relatively short period of five consecutive working days per ward. Therefore, the learning curve of prescribers on detecting DRPs cannot be taken into account. Secondly, only pharmacists detected the DRPs and analyzed the severity of the DRP, while physicians might rate the impact of the DRP differently. Also, the perspective of the patient on the DRPs was not taken into account, for example the effect on quality of life. We suggest that future studies focus on the effects of medication reviews on the impact of quality of life by also including the patients perspective. Thirdly, for our cost–benefit analysis indirect and opportunity costs were not included in this study. We believe that including these costs in the cost–benefit analysis will still lead to a positive cost–benefit ratio. Finally, this is a single center study in a university hospital, therefore the results should be interpreted with care regarding other hospitals settings. However, our results can be generalized in the Netherlands, since all hospitals use CDSS based on the Dutch national drug database G-standard. Due to detection of many relevant DRPs in our study, we encourage hospital pharmacist to implement hospital-wide medication reviews.

## Conclusion

Hospital-wide medication reviews by clinical pharmacists have a positive cost–benefit ratio and contribute to the detection and the resolution of DRPs, mainly by reducing overtreatment.

## Supplementary Information

Below is the link to the electronic supplementary material.

Supplementary file1 (DOCX 15 kb)
