# Supplementary material for: A cost–benefit analysis of hospital-wide medication reviews: a period prevalence study
Source: Int J Clin Pharm. 2021 Sep 8;44(1):138–45. doi: 10.1007/s11096-021-01323-1 (PMC8866269; doi:10.1007/s11096-021-01323-1)
Supplement: Supplementary file 1 — Supplementary file1 (DOCX 15 kb) [file 11096_2021_1323_MOESM1_ESM.docx]

**Supplementary Information (SI)**

**Title**

A cost-benefit analysis of hospital-wide medication reviews: a period prevalence study

**Journal**
International Journal of Clinical Pharmacy

**Author information**

Sarah Wilkes^1^; Rianne Zaal^1^; Alan Abdulla^1^; Nicole Hunfeld^1,2^

1. Erasmus MC, University Medical Center Rotterdam, Department of Hospital Pharmacy. The Netherlands.
2. Erasmus MC, University Medical Center Rotterdam, Department of Intensive Care. The Netherlands.

**Corresponding author**S. Wilkes

E-mail address: s.wilkes@erasmusmc.nl

**S1.** Overview of the clinical wards included in the study

| **Adult clinic** | **Children’s hospital** |
| --- | --- |
| Intensive care unit | Neonatology intensive care unit |
| Cardiac intensive care unit | Children’s intensive care unit |
| Oncology | Oncology |
| Hematology | Hematology |
| Orthopedic and surgical | Orthopedic and surgical |
| Internal medicine (e.g. nephrology, dermatology) | Internal medicine (e.g. nephrology, dermatology) |
| Gastroenterology | Gastroenterology |
| Psychiatry | Psychiatry |
| Neurology and neurosurgery | Neurology and neurosurgery |
| Cardiology | Cardiology |
| Pulmonary | Pulmonary |
| Obstetrics | Urology |
| Gynecology and urology |  |
